# Pancreatic Metastasis of Renal Cell Carcinoma: A Surgical Indication for a Disseminated Disease

**DOI:** 10.1155/2021/5579385

**Published:** 2021-03-06

**Authors:** Feryel Letaief Ksontini, Salim Khrouf, Selma Kacem, Anis Haddad, Houcine Magherbi, Youssef Chaker, Mouna Ayadi, Zoubair Ben Safta

**Affiliations:** ^1^Medical Oncology Department, Salah Azaiz Institute, 1002 Bab Saadoun, Tunis, Tunisia; ^2^Surgical Oncology Department, Salah Azaiz Institute, 1002 Bab Saadoun, Tunis, Tunisia; ^3^General Surgery Department, La Rabta Hospital, 1002 Bab Saadoun, Tunis, Tunisia

## Abstract

Pancreatic metastasis (PM) of renal cancer is a rare condition. It is characterized by a long period after initial nephrectomy and a favorable prognosis compared to other pancreatic malignancies. Its diagnosis may confuse clinicians if the medical history is not known. In the era of targeted therapies for metastatic renal carcinoma, surgery stands as the best treatment option for PM of renal cancer. We report the case of a woman who underwent successfully left splenopancreatectomy for corporeal PM of renal cancer treated seven years ago. This case underlines the necessity of long-term follow-up of patients treated for kidney cancer.

## 1. Introduction

According to the GLOBOCAN database, 403000 people were diagnosed with kidney cancer during 2018. Among them, men were more hit (254000 new cases and 114000 deaths) than women (149000 new cases and 61000 deaths) [1]. Renal cell carcinoma represents 3–5% of all adult malignancies [2], of which clear cell carcinoma represents the most frequent subtype (65–70%) [3].

In Tunisia, it is a less frequent cancer with 2.2 per 100000 incidence [4]. Pancreatic metastasis (PM) of renal cancer is even rarer. Surgical management of metachronous metastasis is a keypoint for this condition. In the present case, we report the case of a patient treated with a left splenopancreatectomy for metachronous pancreatic metastasis, seven years after nephrectomy.

## 2. Case Report

A 60-year-old woman presented in April 2013 with gross hematuria. Physical examination revealed a left flank mass. The abdominal magnetic resonance imaging (MRI) showed a renal tumor measuring 15 × 10 × 12 cm with capsular rupture, invasion of the left renal vein and of the vena cava in which a thrombus extended up to 7 cm. The thoracoabdominal computed tomography (CT) found the same tumor and four nonspecific pulmonary micronodules. This tumor was classified T3cN0M0.

The patient underwent a left total nephrectomy in the same month with a good clinical course. Histologic samples confirmed the diagnosis of a clear cell renal carcinoma classified as pT3cN0M0. She was put into surveillance, and annual CT scans confirmed the stability of the pulmonary nonspecific micronodules with no distant metastases.

After seven years, in November 2020, she consulted for abdominal pain without any other signs. The patient was in a good general condition. The physical examination was unremarkable.

An abdominal sonography was performed revealing a deep hypoechogenic mass of the left flank linked to the tail of the pancreas measuring 7 × 4 cm with irregular outline and extending to the left border of the aorta.

**Figure 1:**
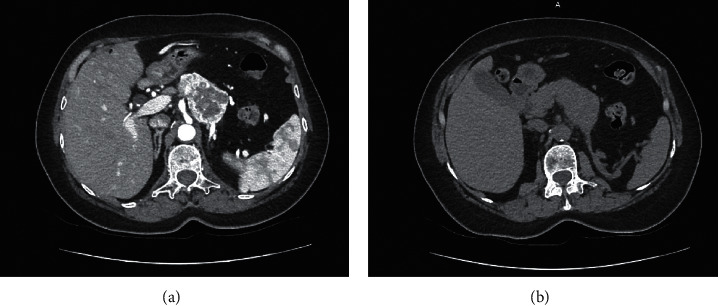
Computed tomography images of a tissular corporeal pancreatic mass, enhanced in periphery (a) with a central necrosis, invading the splenic vein (a, b).

**Figure 2:**
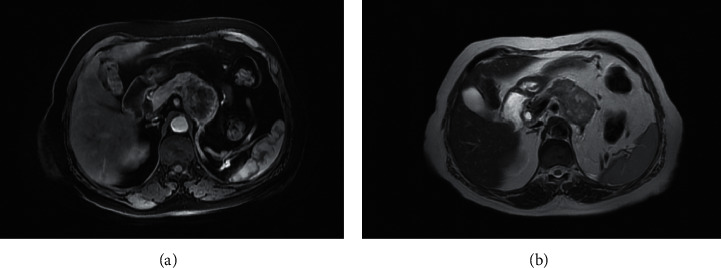
MRI images of T1 fat saturation sequences with gadolinium injection in arterial (a) and portal (b) phases showing a tissular corporeal pancreatic mass, with low signal, enhanced in periphery, comprising a necrotic center with no involvement of the superior mesenteric artery.

**Figure 3:**
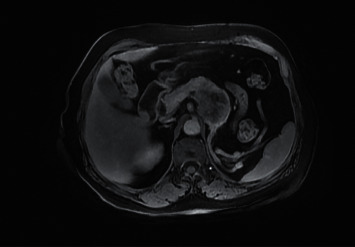
MRI T2 images of a heterogeneous hypersignal corporeal pancreatic mass.

The abdominal CT scan showed a multilobular mass, hypervascular on the arterial phase with arteriovenous fistula and wash-out on the venous phase. This mass obstructed the splenic vena and was responsible of a collateral venous circulation as well as of the atrophy of the tail of the pancreas (Figure 1). An abdominal MRI confirmed the same findings with a corporeal mass that had low signal in T1 Fat Sat images and hyperintense signal in T2 images (Figures 2 and 3). No further locations were found. A thoracic scan was performed and did not show any abnormality.

After discussion of this case in a multidisciplinary meeting, a left splenopancreatectomy was performed. Surgical samples showed a 50 × 40 mm red-whitish nodular tumoral neoformation of the tail and body of the pancreas with some necrotic changes. Postoperative recovery was eventful.

**Figure 4:**
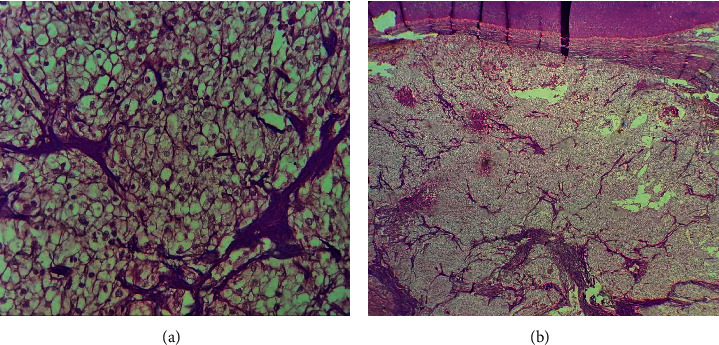
(a) (GX400) Clear cell carcinoma with a regular network of small and thin walled blood vessels. (b) (Gx200) Clear cell carcinoma with solid alveolar arrangement of cells.

The histological examination revealed a malignant proliferation in the pancreatic parenchyma made of cordonal, trabecular, and alveolar anastomotic structures. These structures were arranged into a fibrohyaline stroma comprising a rich vascular network, punctuated by an inflammatory infiltrate with punctiform necrotic changes. Tumoral cells possess a clear cytoplasm. Nuclei were often irregular with visible nucleolus. The tumor did not invade the peripancreatic fat. The study concluded to a pancreatic metastasis of renal cell carcinoma without metastatic lymph nodes (Figure 4).

The multidisciplinary meeting decided to continue surveillance without any further adjuvant treatment.

One month after surgery, the patient did not show any relapse and was relieved from its symptoms.

## 3. Discussion

In renal cell carcinoma, major metastatic sites are the lungs (45.2%), bones (29.5%), lymph nodes (21.8%), liver (20.3%), and adrenals (8.9%) [5]. However, despite pancreatic involvement is not frequent, RCC is the most common primary tumor leading to pancreatic metastasis [5].

Pancreatic metastasis (PM) of renal cell carcinoma (RCC) incidence in autopsy series is reported between 1% and 3% [6]. They are also a rare pancreatic disease and represent about 2% of all pancreatic malignancies [7].

Epidemiologic data on primary tumor location (left or right kidney) and PM location on the pancreas found no correlation and pleaded in favor of a hematogenous dissemination of the disease rather than a lymphogenous local spread [8]. By a biochemical phenomenon of tumor cell selection, emboli metastatic cells only develop in the pancreatic parenchyma but not in other organs, explaining why PM is usually unique [9]. Later developing metastases in remote organs are due to the heterogeneity of the primary tumor, which spreads genetically different cells [8].

Clinically, pancreatic metastases are usually asymptomatic as 55% of them show no symptoms and are discovered in surveillance imaging [7]. Very often, this condition is discovered a long period after initial nephrectomy, at a median interval of 91 months (54–142) [10]. This finding underlines the necessity of long-time follow-up of renal cancer patients. Metachronous disease is rarer, and its incidence is estimated at 12% [11].

When symptomatic, patients may experience nausea, jaundice, acute pancreatitis, abdominal pain, gastrointestinal bleeding, or weight loss [6].

PM is characterized in computed tomography (CT) by its isodensity or hypodensity unenhanced images and by its avidity for enhancement in the late arterial phase as well as a wash-out in late phases [12]. Similar findings were described on MRI, lesions having low signal in T1 images and are hyperintense in T2 images [12].

Pancreatic lesions are more often multiple when they are secondary to kidney cancer [13], and the multifocal presentation of pancreatic metastases to kidney cancer represents between 20% and 45% of cases [14].

Multifocality and the pattern of enhancement are the key findings that differentiate primary from secondary pancreatic lesions.

Some PMs have an intense octreotide uptake in scintigraphy which may distort the diagnosis, confusing with neuroendocrine tumours [15]. However, usually, these tumours do not invade neither the choledochus nor the pancreatic duct [16, 17].

The surgical management of metastatic renal cell cancer has changed in recent years with the development of target therapies and immunotherapy. Resection of metachronous metastases is more commonly described than synchronous metastases. For metachronous metastases, it is now consensual that surgery is the optimal treatment [11]. In a series of 274 patients with PM (both metachronous and synchronous), Grassi et al. found that patients locally treated had a doubled median OS compared with patients with systemic treatment with targeted therapies [10]. Atypical resection is preferred to typical techniques because oncologic results are comparable when removing only the metastasis and protecting the endocrine and exocrine function [18]. The decision of surgery instead of systemic drugs is motivated by an excellent prognosis of these patients as overall survival at 5 years is estimated at 72.6% in a meta-analysis of 311 patients with PM [19]. It is suggested that patients suffering from PM of RCC present a less aggressive disease. Kalra et al. studied retrospectively 228 metastatic RCC in both PM and non-PM patients. The PM cohort was associated with a better prognosis than the other cohort, with a similar otherwise metastatic distribution [20]. Very few cases have been reported of surgery performed for synchronous disease, but the surgical management of these lesions is still under debate. Nevertheless, it should be performed along with nephrectomy, whenever the patient is fit, and PM, the only site involved, as a persistent kidney tumor may be a source of potential new metastases [21].

The place of targeted therapies remains uncertain, as no data are currently available on their usefulness in patients with PM alone. The good results of surgery in terms of overall survival lead us to consider their use only after surgical treatment [22].

Prognostic factors reported in the literature are symptomatic metastases, surgical intervention [18], nephrectomy, and good or intermediate Memorial Sloan-Kettering Cancer Center (MSKCC) score that were related to survival [10].

In summary, metachronous PM of RCC occurs late in the disease natural history, highlighting the necessity of long-term surveillance. It is associated with a good prognosis, and its treatment is based on the surgery of the metastases.

However, it is important to highlight that more than 20% of patients have metastatic disease at presentation, and early screening is necessary. Many studies suggest different imaging techniques, while others are developing the molecular diagnosis with detection of urinary miRNAs as a diagnostic tool of clear cell renal cell carcinoma [23].

## Data Availability

The data used to support the findings of this study are included within the article.
